# Study on Different Molecular Weights of Chitosan as an Immobilization Matrix for a Glucose Biosensor

**DOI:** 10.1371/journal.pone.0070597

**Published:** 2013-08-05

**Authors:** Lee Fung Ang, Lip Yee Por, Mun Fei Yam

**Affiliations:** 1 School of Pharmaceutical Sciences, Universiti Sains Malaysia, Minden, Penang, Malaysia; 2 Faculty of Computer Science and Information Technology, University of Malaya, Kuala Lumpur, Malaysia; National University of Ireland, Galway, Ireland

## Abstract

Two chitosan samples (medium molecular weight (MMCHI) and low molecular weight (LMCHI)) were investigated as an enzyme immobilization matrix for the fabrication of a glucose biosensor. Chitosan membranes prepared from acetic acid were flexible, transparent, smooth and quick-drying. The FTIR spectra showed the existence of intermolecular interactions between chitosan and glucose oxidase (GOD). Higher catalytic activities were observed on for GOD-MMCHI than GOD-LMCHI and for those crosslinked with glutaraldehyde than using the adsorption technique. Enzyme loading greater than 0.6 mg decreased the activity. Under optimum conditions (pH 6.0, 35°C and applied potential of 0.6 V) response times of 85 s and 65 s were observed for medium molecular weight chitosan glucose biosensor (GOD-MMCHI/PT) and low molecular weight chitosan glucose biosensor (GOD-LMCHI/PT), respectively. The apparent Michaelis-Menten constant (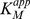) was found to be 12.737 mM for GOD-MMCHI/PT and 17.692 mM for GOD-LMCHI/PT. This indicated that GOD-MMCHI/PT had greater affinity for the enzyme. Moreover, GOD-MMCHI/PT showed higher sensitivity (52.3666 nA/mM glucose) when compared with GOD-LMCHI/PT (9.8579 nA/mM glucose) at S/N>3. Better repeatability and reproducibility were achieved with GOD-MMCHI/PT than GOD-LMCHI/PT regarding glucose measurement. GOD-MMCHI/PT was found to give the highest enzymatic activity among the electrodes under investigation. The extent of interference encountered by GOD-MMCHI/PT and GOD-LMCHI/PT was not significantly different. Although the Nafion coated biosensor significantly reduced the signal due to the interferents under study, it also significantly reduced the response to glucose. The performance of the biosensors in the determination of glucose in rat serum was evaluated. Comparatively better accuracy and recovery results were obtained for GOD-MMCHI/PT. Hence, GOD-MMCHI/PT showed a better performance when compared with GOD-LMCHI/PT. In conclusion, chitosan membranes shave the potential to be a suitable matrix for the development of glucose biosensors.

## Introduction

A biosensor is commonly described as an analytical device incorporating a biological or biologically derived recognition element, either intimately associated or integrated within a physicochemical transducer to produce a signal proportional to the target analyte concentration [1]. What is important is a direct relationship between the biosensor signal and the quantity of the analyte. Since the invention of the first oxygen electrode by Clark and Lyons (1962), enzymes have been the most regularly employed biorecognition elements encountered in catalytic biosensors for the analysis of small molecules such as glucose, which is widely monitored in medicine, biotechnology and the food industry [2], [3].

Glucose biosensors are categorized based on the type of transducer used, such as electrochemical, piezoelectric, thermoelectric, acoustic and optical sensors (Wilkins &Atanasov, 1996) [4]. In electrochemical sensors, the electrical signal is a direct result of a chemical process occurring at the transducer/analyte interface [4]. Electrochemical transducers include potentiometric, voltammetric (amperometric), conductometric and field-effect transistor (FET)-based electrodes [5]. To date, electrochemical sensors dominate in glucose sensing owing to the simplicity of electrochemical measuring principles and the low cost [5]. Glucose oxidase was selected as a model enzyme in this study. Glucose oxidase is highly specific for β-D-glucose. It is widely used for the determination of glucose in body fluids and in removing residual glucose and oxygen from beverages and foodstuffs. Moreover, glucose oxidase is suitable for biosensor applications.

Chitosan was selected as a matrix for immobilization of the enzyme because of its biocompatibility, non-toxicity, high mechanical strength and excellent membrane forming ability. Chitosan can be divided into three categories, namely low molecular weight, medium molecular weight and high molecular weight. Chitosan of higher molecular weight possesses longer molecular chains with the availability of more hydroxyl groups. There is also a higher possibility that there are more amino groups, although the number of amino groups is determined by the degree of deacetylation. These amino groups are responsible for crosslinking. However, a review of the current literature revealed leaked documented reports on a comparative study of differences in the molecular weight of chitosan as enzyme matrix for biosensor. Therefore, in the present study, we investigated whether higher molecular weight chitosan could improve enzyme retention activity and loading, and thus function as a suitable matrix for enzyme immobilization. By using this chitosan membrane as a biosensor, it was hypothesized that it would provide a better performance in terms of sensitivity and stability. In this study, we report the development, fabrication and characterization of an amperometric-based glucose biosensor with the aim of comparing the effectiveness of chitosan of different molecular weights as a matrix for enzyme immobilization using adsorption and crosslinking techniques, and to study the behavior of these enzyme-chitosan electrodes.

## Results and Discussion

### Investigation of the Intermolecular Interactions of Immobilized GOD-chitosan Membranes using FTIR

**Figure 1:**
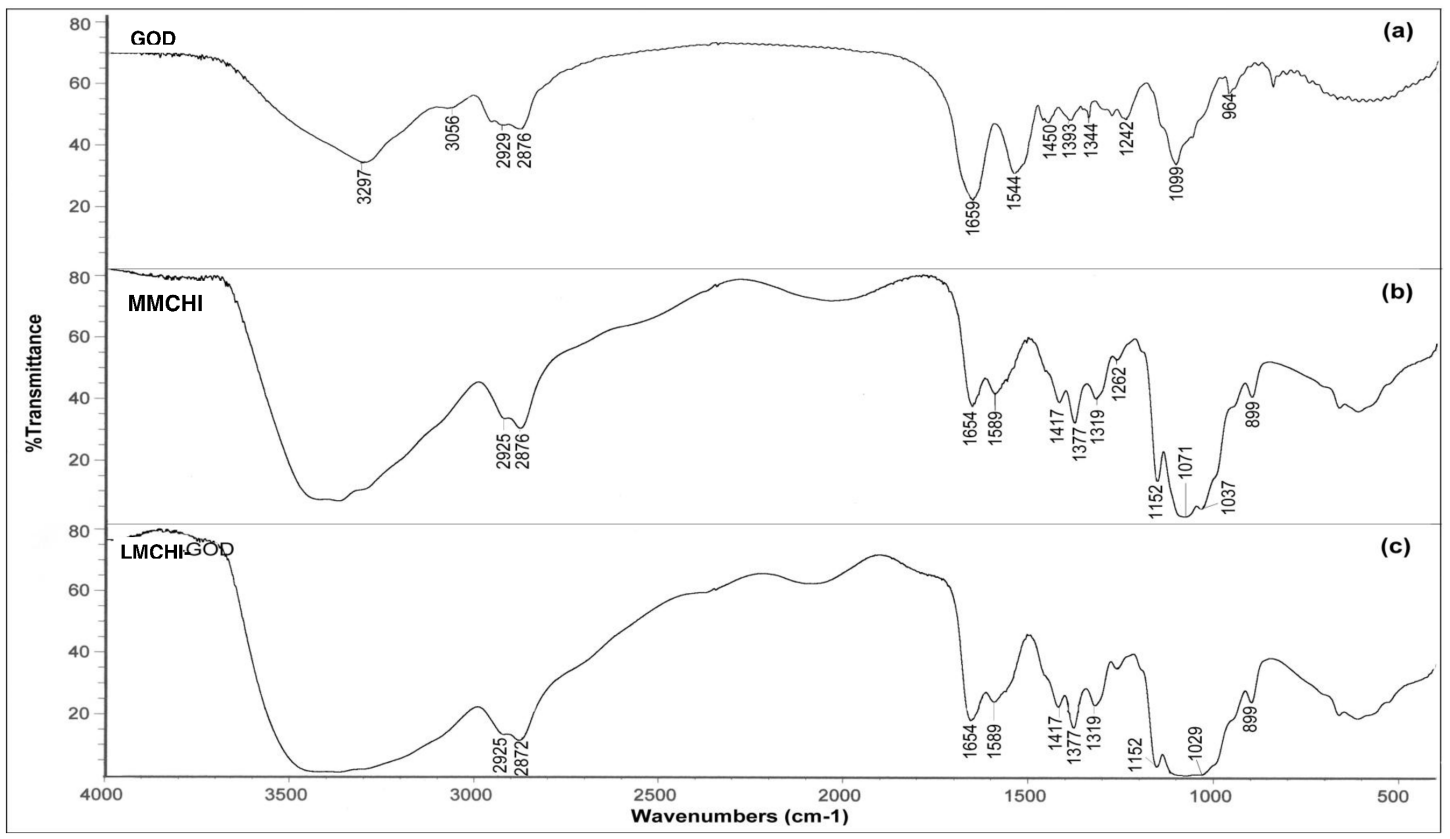
FTIR spectra of (a) crystalline GOD; (b) MMCHI membrane; (c) GOD-MMCHI membrane showing the interactions between GOD and chitosan membrane after immobilization.

FTIR is a powerful tool for identifying the types of chemical bonds in a molecule by producing an infrared absorption spectrum that is like a “molecular fingerprint”. In this study, FTIR spectroscopy was used to investigate the interactions between chitosan and glucose oxidase. Figure 1 displays the Fourier transform infrared spectroscopy (FTIR) spectra of glucose oxidase (GOD) crystals, medium molecular weight chitosan (MMCHI) membrane and the GOD-MMCHI membrane.

Detailed information on the secondary structure of a polypeptide chain is provided by the shape of the amide I and amide II infrared absorbance bands of the protein [6]. In the crystalline GOD spectrum, the C = O stretching of the amide bond was observed at 1659 cm^−1^. The peak at 1544 cm^−1^ was assigned to strong N-H bending vibration of the secondary amide [7]. The absorption bands between 1340 cm^−1^ and 1450 cm^−1^ were attributed to symmetric and asymmetric vibrations of COO^−^ groups [8]. The bands for symmetric and asymmetric vibrations of CH_2_ groups were found at 2876 cm^−1^ and 2929 cm^−1^, respectively [1], [9]. The strong band observed at 3297 cm^−1^ and a weaker band at 3056 cm^−1^were attributed to amide A and amide B bands of GOD, respectively. The amide A band arises due to N-H stretching vibrations, whereas the amide B band arises due to the first overtone of the amide II vibration intensified by Fermi resonance with amide A vibrations [1].

**Figure 2:**
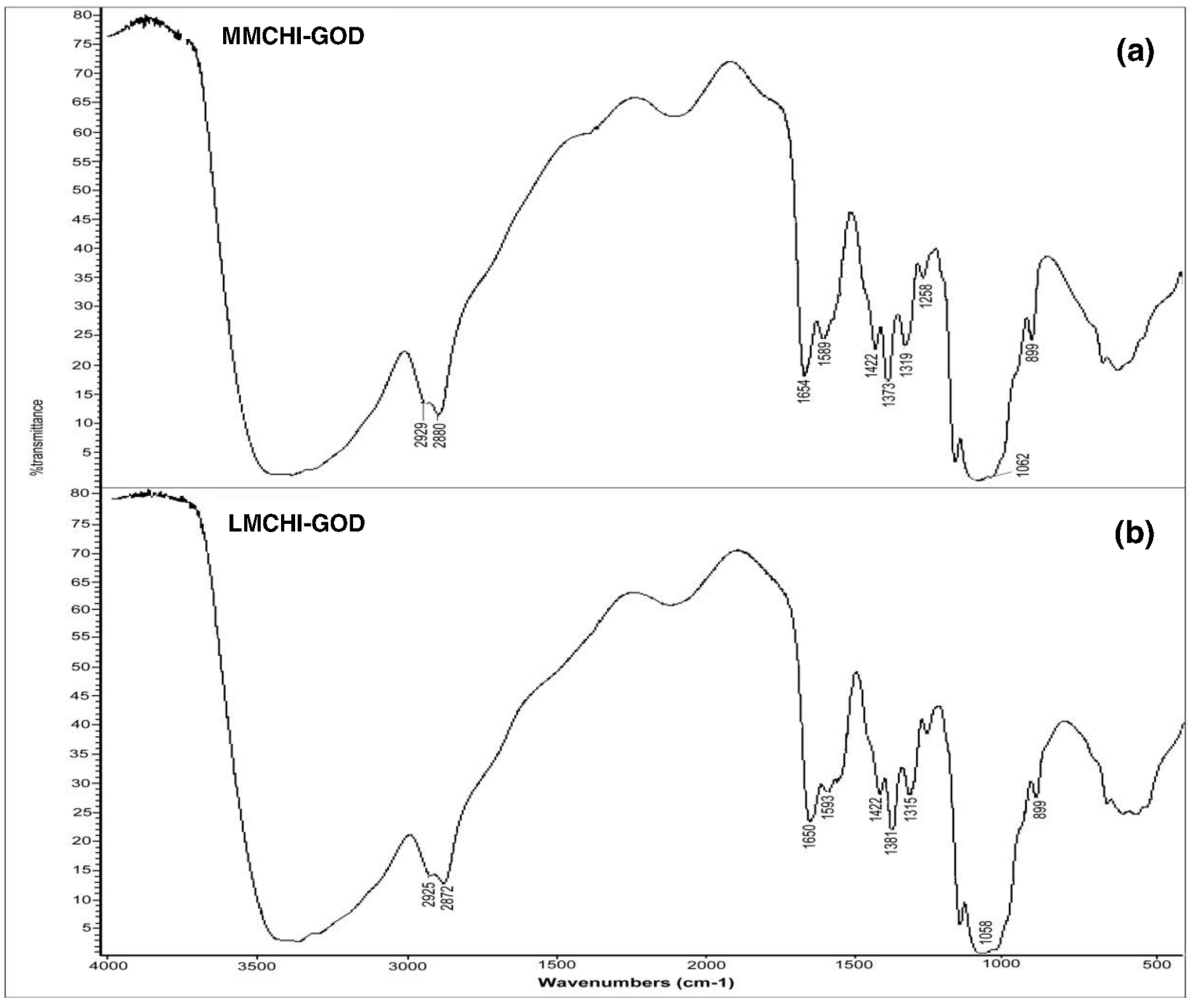
FTIR spectra of (a) GOD-MMCHI membrane and (b) GOD-LMCHI membrane.

Although the overall FTIR spectra of the MMCHI membrane (cast from acetic acid), crystalline GOD and the GOD-MMCHI membrane were similar to each other, they showed subtle differences in the absorption intensities and range of frequencies. The presence of residual moisture content and glycerol in the GOD-MMCHI membrane resulted in a broad peak from 3300 to 3500 cm^−1^. The amide I and II bands of the GOD-MMCHI membrane increased in intensity compared to that of the MMCHI membrane. The peak at 1544 cm^−1^ in the spectrum of crystalline GOD shifted to 1589 cm^−1^ in the GOD-MMCHI membrane. This could be due to intermolecular interactions between the enzyme and membrane via the formation of a Schiff base linkage between the aldehyde groups of glutaraldehyde and the amine groups of GOD and chitosan. Musale and Kumar (2000) reported that increased intensity of an amide II band may be due to the overlapping of peaks corresponding to -N-H stretching in –NHCOCH_3_ of chitosan and C = N stretching of the newly formed Schiff base between the –NH_2_ groups of chitosan and the  = CHO groups of glutaraldehyde [10]. In the spectrum of the GOD-MMCHI membrane, the heme vibrational modes (1340–1400 cm^−1^) [11] of GOD are overlapped with the bands of CH_3_ bending of the MMCHI membrane. The 1000–1100 cm^−1^ region in the GOD-MMCHI membrane spectrum appeared as a characteristic absorption spectrum typical of the phosphate buffer in the enzyme solution. Furthermore, the broad band of O-H stretching resulted from intermolecular hydrogen bonds between chitosan and GOD molecules. Figure 2 shows the FTIR spectra of the GOD-MMCHI membrane and the GOD-LMCHI (low molecular weight chitosan) membrane displaying a similar pattern.

### Catalytic Activity Measurements of the Soluble and Immobilized Enzyme

The GOD-peroxidase (POD) enzymatic system is an assay for determining glucose concentrations based on a spectrophotometric method. In the catalyzed reaction of GOD, H_2_O_2_ is produced as a side product, which is then reduced in the POD enzymatic reaction. 2,2′-azino-di-(3-ethylbenzthiazoline)-6-sulfonic acid (ABTS) was used as a hydrogen donor in the H_2_O_2_ reduction process. It was oxidized to a stable, non-toxic, water-soluble brilliant blue-green derivative (ABTS^+^). The intensity of the solution color is proportional to the ABTS^+^ concentration and indirectly to the H_2_O_2_ concentration. The amount of ABTS^+^ generated was also a measure of the glucose oxidized with two moles of ABTS oxidized per mole of glucose.

The molar extinction coefficient of flavin adenine dinucleotide (FAD) permits direct a calculation of the concentration of H_2_O_2_ produced from the absorbance as a function of time plot. The angular coefficient of the straight line at the initial rates of H_2_O_2_ production gives the enzyme activity expressed as µmol/min. In this way, the specific enzyme activity (SEA) in units/mg can be calculated using the following equation:
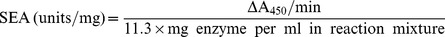
where 11.3 refers to the millimolar extinction coefficient of oxidized ABTS at 450 nm. One unit (U) of activity of glucose oxidase is defined as the amount of enzyme that catalyzes the oxidation of one µmol of ABTS per minute under the above mentioned conditions. The retention activity of the immobilized enzyme was then obtained by expressing the SEA of immobilized enzyme as a percentage of the SEA of the soluble enzyme (Table 1).

**Table 1:** Catalytic activity of different immobilized enzyme-membranes.

Amount of GOD (mg)	Specific enzyme activity (Units/mg)					Retention activity (%)			
	Soluble GOD	cGOD-MMCHI	cGOD-LMCHI	AGOD-MMCHI	AGOD-LMCHI	cGOD-MMCHI	cGOD-LMCHI	AGOD-MMCHI	AGOD-LMCHI
0.05	1.402±0.002	1.042±0.007	0.964±0.007	0.566±0.002	0.525±0.002	74.32	68.76	40.37	37.45
0.10	1.174±0.003	0.901±0.006	0.857±0.005	–	–	76.75	73.00	–	–
0.20	1.025±0.002	0.810±0.007	0.789±0.006	–	–	79.02	76.98	–	–
0.40	0.737±0.001	0.623±0.004	0.599±0.003	–	–	84.53	81.28	–	–
0.60	0.622±0.003	0.621±0.003	0.561±0.003	–	–	99.84	90.19	–	–
0.80	0.482±0.002	0.429±0.001	0.416±0.002	–	–	89.00	86.31	–	–

Mean±S.E.M, n = 3.

c Enzyme immobilized on chitosan membrane via glutaraldehyde crosslinking.

A Enzyme immobilized on chitosan membrane by physical adsorption.

The most important aspect of enzyme immobilization is the retention of its activity upon immobilization. As shown in the tabulated results, the immobilized enzyme retained a relatively high activity with varying amounts of enzyme for the crosslinking method employed, presumably due to strong crosslinking with the formation of a Schiff base linkage. MMCHI membranes showed slightly higher activity when compared to LMCHI membranes. This might be due to the higher molecular weight (MW) of MMCHI. In a study by Xu and Du (2003) on the molecular structure of chitosan, they reported that chitosan with higher MW possess longer polysaccharide chains which can entrap a greater amount of protein [12]. Alsorra *et al.* (2002) reported that chitosan with high MW could improve enzyme loading and reduce enzyme release [13]. In the present study, MMCHI with higher MW showed a higher retention activity as a result of greater enzyme loading. On the other hand, the weak binding of the enzyme to the matrix in the adsorption method resulted in low enzyme activity.

**Figure 3:**
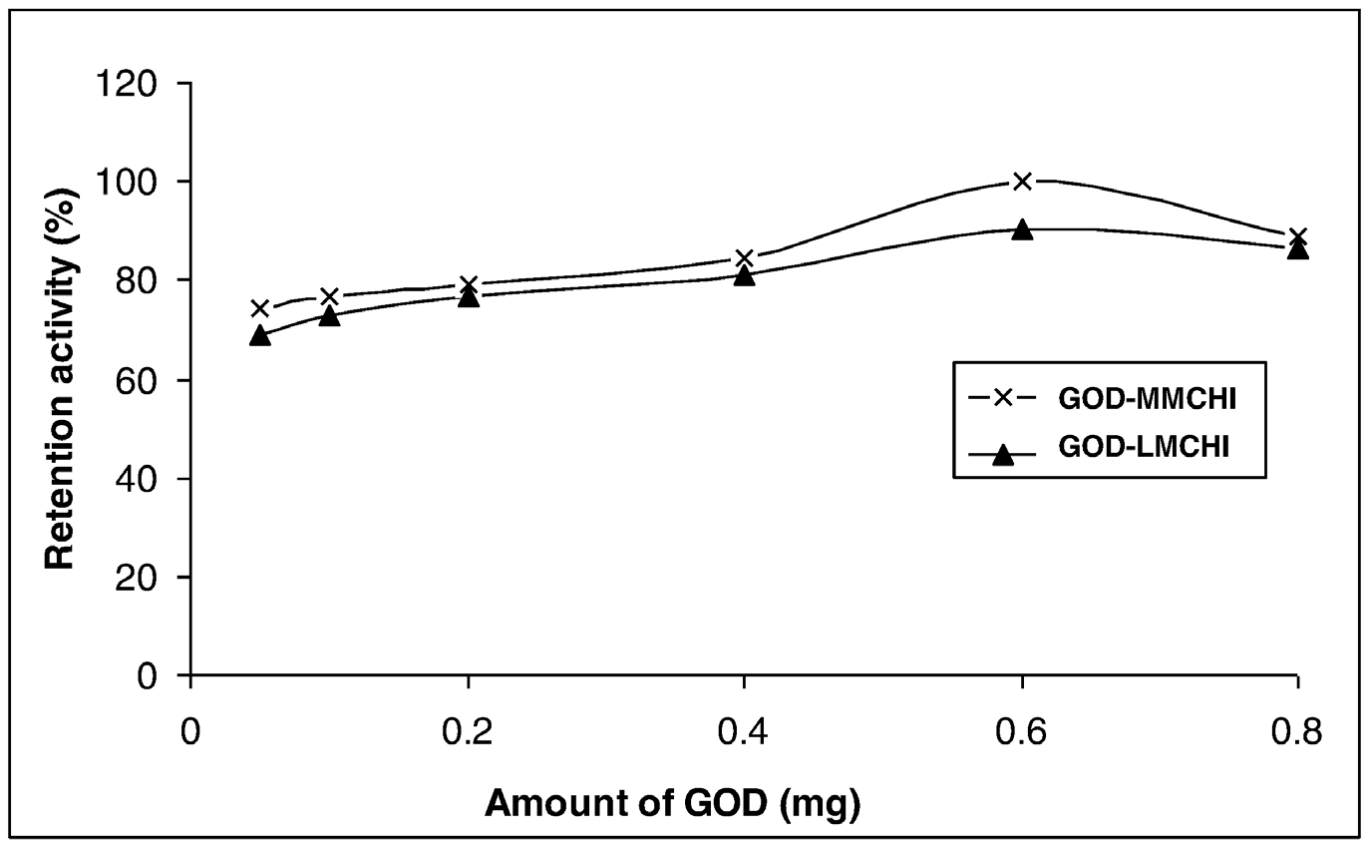
Effect of enzyme loading on the retention activity of GOD on chitosan.

The effect of enzyme loading on the activity of the immobilized enzyme (via glutaraldehyde crosslinking) was studied by varying the amount of GOD (2.5–40.0 mg/mL or 0.05–0.80 mg) for immobilization (Figure 3). The enzymatic activity increased with increasing the amount of GOD up to 0.6 mg (30.0 mg/mL), but decreased at higher GOD content. Similar results were reported by Onda *et al*. (1999) and Bindhu & Abraham (2003) [14], [15]. The drop in enzyme activity at high enzyme concentrations might be due to the increased hindrance in substrate diffusion on the surface of the membrane.

### Determination of the Michaelis-Menten Constant for the Soluble Enzyme

**Figure 4:**
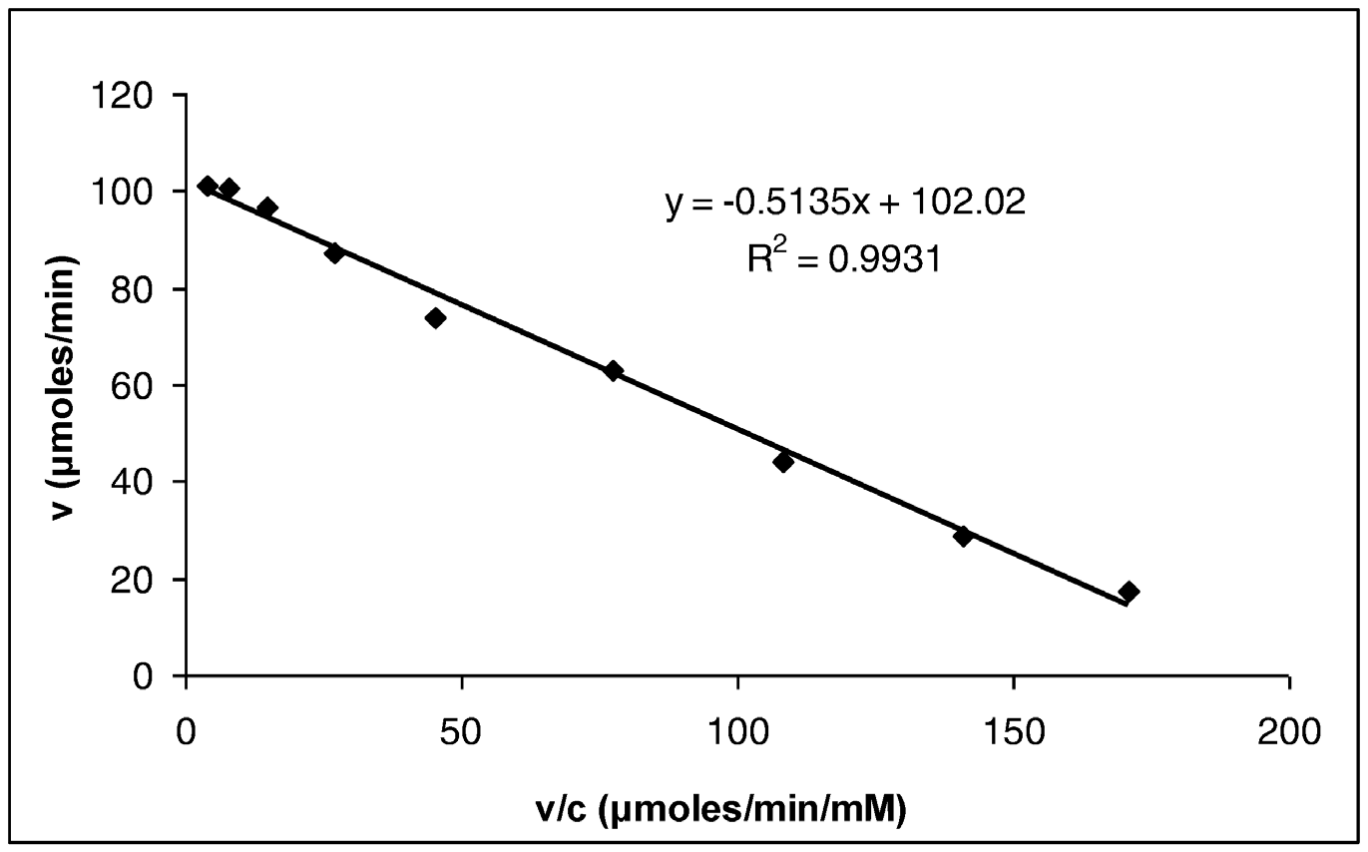
Eadie-Hofstee plot for the determination of Michaelis-Menten constant of the soluble GOD.

The Michaelis-Menten constant, 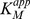 for the soluble GOD determined from Eadie-Hofstee plot (Figure 4) was found to be 0.5135 mM, indicating a strong affinity between enzyme and substrate.

### Characteristics of the Glucose Biosensor

#### Response time

**Figure 5:**
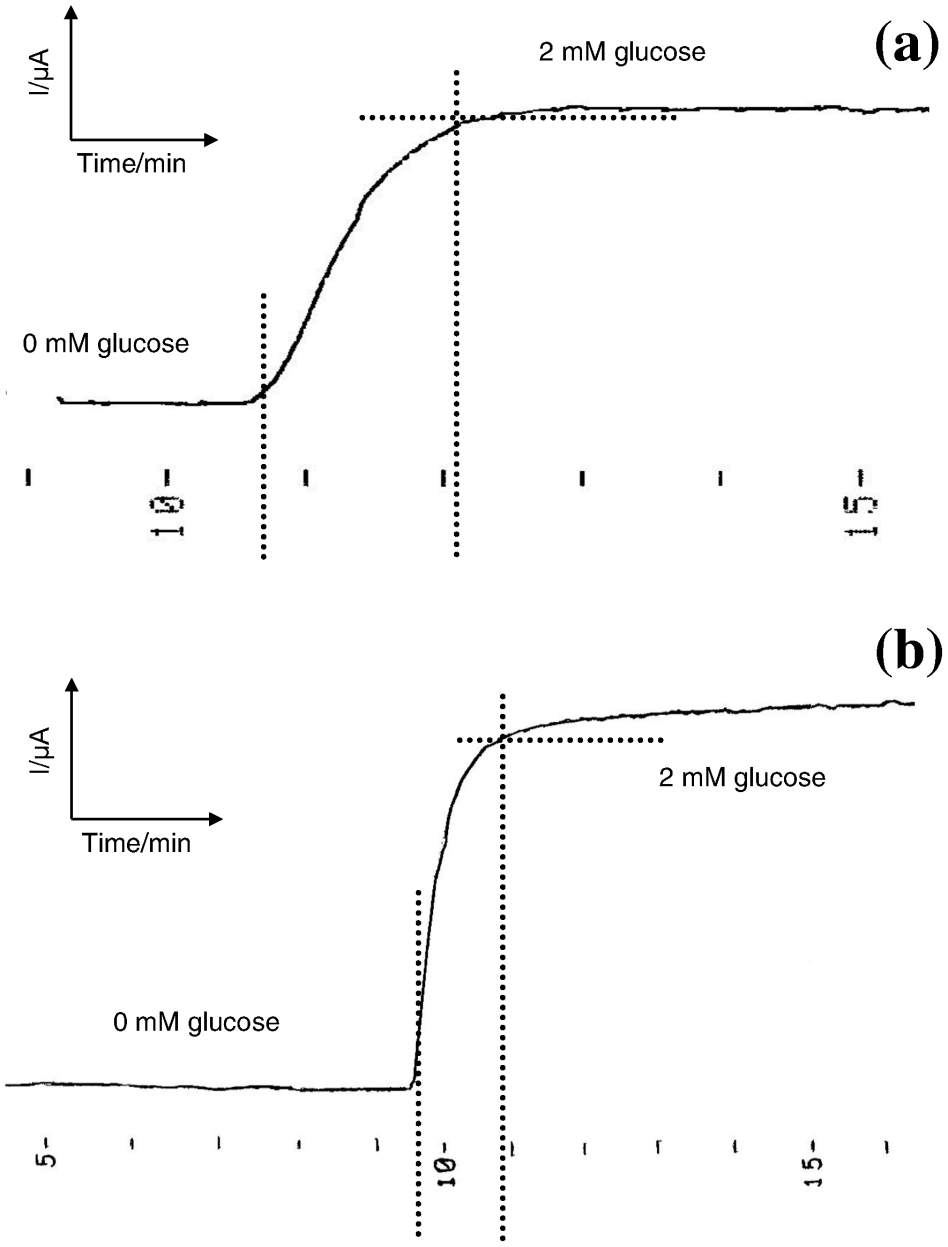
(a)Response time curve for GOD-FCHIT/PT to glucose; (b) Response time curve for GOD-SCHIT/PT to glucose.

The response time is defined as the time required to reach a steady-state current value on sensing the substrate. Figures 5, shows that the response times of medium molecular weight chitosan glucose biosensor (GOD-MMCHI/PT) and low molecular weight chitosan glucose biosensor (GOD-LMCHI/PT) to 2 mM glucose were less than 85 s and 65 s, respectively. The fast response time indicated good permeability of the chitosan membrane in the biosensors to the enzymatically-generated H_2_O_2_.

### Calibration of Glucose Biosensor

**Figure 6:**
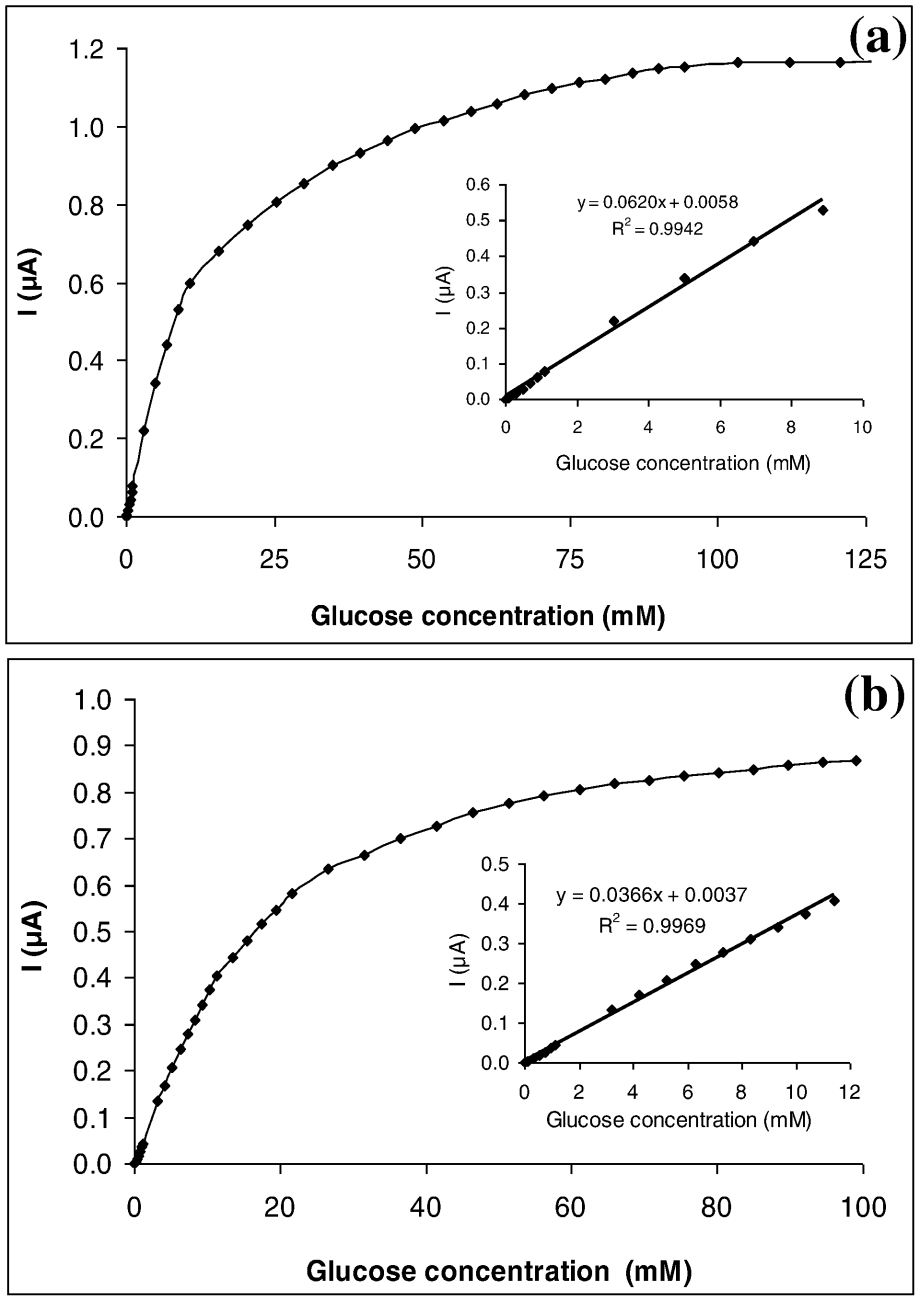
(a) Calibration curve of the GOD-FCHIT/PT under optimal experimental conditions. Inset: Linear range from 10.0 µM to 10.8 mM glucose with linear regression equation y = 0.0620*x* +0.0058; R^2^ = 0.9942, n = 6.(b) Calibration curve of the GOD-SCHIT/PT under optimal experimental conditions. Inset: Linear range from 10.0 µM to 11.4 mM glucose with linear regression equation y = 0.0366*x* +0.0037; R^2^ = 0.9969, n = 5.

GOD-MMCHI/PT and GOD-LMCHI/PT were calibrated under optimal experimental conditions with glucose concentrations from 9.99×10^−6^ to 1.29×10^−1^ M and 1.00×10^−5^ to 9.90×10^−2^ M, respectively (Figures 6). The anodic current increased with increasing glucose concentration. The GOD-MMCHI/PT biosensor exhibited a linear calibration curve up to 10.8 mM. The slope of the initial range was 0.0620 µA/mM with a correlation coefficient of 0.9942 (Figure 6 (a), inset). A detection limit (S/N>3) of 0.1 mM glucose with a sensitivity of 52.3666 nA/mM was obtained. In the case of GOD-LMCHI/PT, the linear range was from 10.0 µM to 11.4 mM with a slope of 0.0366 µA/mM and a correlation coefficient of 0.9969 (Figure 6 (b), inset). The detection limit of 0.1 mM glucose at S/N>3 with a sensitivity of 9.8579 nA/mM was obtained.

Singhal *et al.* (2002) reported that the normal blood glucose concentration for humans is in the range of 80 to 140 mg/dL (4.44–7.77 mM). In the present study, both biosensors exhibited a wide linear range covering the range of normal blood glucose concentrations [1]. GOD-MMCHI/PT showed a greater sensitivity in the amperometric measurement when compared to GOD-LMCHI/PT. The limit of detection of GOD-MMCHI/PT was lower compared to GOD-LMCHI/PT, indicating that GOD-MMCHI/PT was more sensitive in the detection of glucose.

### Determination of the Apparent Michaelis-Menten Constant

**Figure 7:**
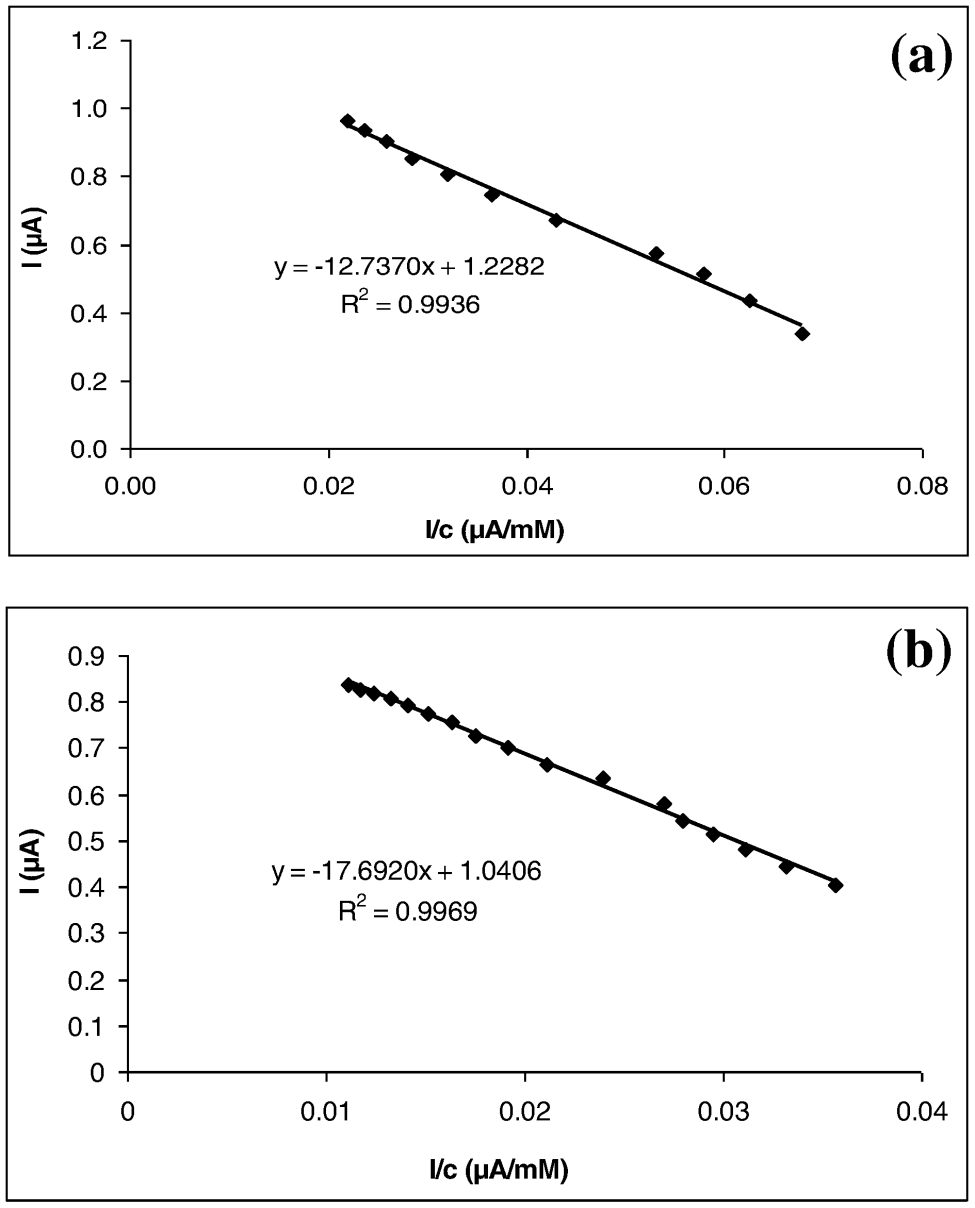
Eadie-Hofstee plot of (a) GOD-MMCHI/PT (b) GOD-LMCHI/PT. The glucose concentration range chosen was optimal for the determination of 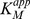 and *I*_max._

The value of the apparent Michaelis-Menten constant (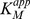) for an enzyme is an indication of its affinity for the substrate; the lower the value, the higher the affinity for immobilized GOD [16].The 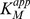 for the immobilized enzyme can be determined by amperometry as suggested by Shu and Wilson (1976) [17]. A linear line was obtained in the Eadie-Hofstee plot for GOD-MMCHIT/PT from 4.99 to 44.17 mM glucose (Figure 7). The linear regression of the plot was y = −12.7370*x* +1.2282 with R^2^ = 0.9936. Thus, the 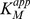 for glucose and *I*_max_ value for the biosensor were found to be 12.7370 mM and 1.2282 µA respectively. Similarly, the Eadie-Hofstee plot for GOD-LMCHIT/PT was also a linear line from 11.39 to 75.47 mM (Figure 7). The linear regression of the plot was y = −17.6920*x* +1.0406 with R^2^ = 0.9969. The 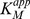 value for GOD was 17.6920 mM and the *I*_max_ was 1.0406 µA.

The smaller the value of 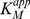, the stronger the affinity between the enzyme and the substrate [18]. The smaller 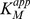 value of GOD-MMCHIT/PT indicated that the immobilized GOD on MMCHIT membrane possessed higher enzymatic affinity. However, when the kinetic parameters of the immobilized GOD were compared to those of soluble GOD (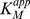 = 0.5135 mM), an increase in 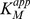 for immobilized GOD was observed. Spagna *et al*. (1997) and Bhatia *et al*. (2000) reported similar behavior for the immobilized enzyme [19], [20]. The increase in 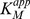 value in the immobilized enzyme could be due to problems associated with the diffusion of the substrate or products through the support [21]. The 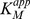 values of both the biosensors were lower when compared to the value of 23.30 mM reported by Chen *et al*. (2006)and 32.71 mM reported by Xu and Chen (2000) [18], [22].

### Repeatability and Reproducibility

A reliable glucose biosensor should show good precision (repeatability and reproducibility). Repeatability refers to the agreement between successive measurements of the same sample whereas reproducibility is used to describe the closeness of agreement between results (signals) obtained with the same method under different conditions (using different glucose biosensors) [23].

Both the glucose biosensors showed both good repeatability and reproducibility in the glucose measurements as indicated by their RSD value. The repeatability and reproducibility of GOD-MMCHI/PT were 2.30% and 3.70% and that of GOD-LMCHI/PT were 4.12% and 9.46%, respectively (Table 2). The precision of the enzyme immobilized on MMCHI was higher compared to that of LMCHI.

**Table 2.The:** repeatability and reproducibility of the biosensors.

Electrodes	Repeatability		Reproducibility	
	I (µA) (mean±SD, n = 20)	RSD (%)	I (µA) (mean±SD, n = 6)	RSD (%)
**GOD-MMCHI/PT**	0.22±0.01	2.30	0.23±0.01	3.70
**GOD-LMCHI/PT**	0.24±0.01	4.12	0.22±0.02	9.46

### Stability Study

**Figure 8:**
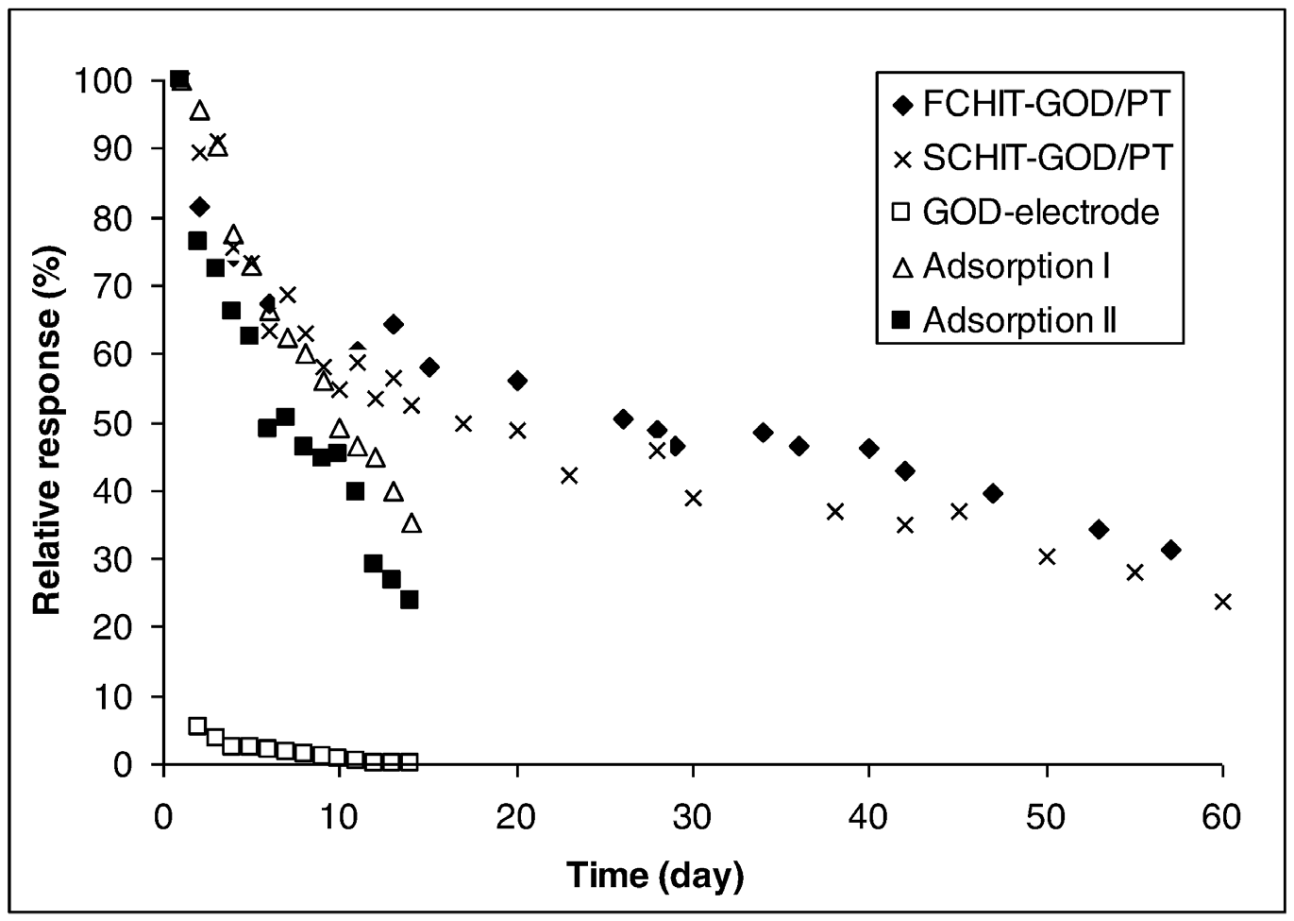
Stability of glucose biosensors over a period of 60 days. Data points shown are the mean value of three biosensors.

Enzyme stability in the matrix is a vital factor to consider in the development of a biosensor. As such, the stability of the GOD bioelectrodes was evaluated over a period of two months (Figure 8).

During the initial period, both GOD-MMCHI/PT and GOD-LMCHI/PT were comparably stable. After one week, both biosensors retained about 60–70% of their original activity. On Day 14, the current was still more than half the initial value. Thereafter, the response dropped gradually to about 20–30% of the initial value at the end of two months. The activity of GOD-MMCHI/PT was higher when compared to GOD-LMCHI/PT throughout the period of investigation. The relatively longer stability of GOD-MMCHI/PT could be due to the higher MW of MMCHI, which could prevent enzyme from leaking [13].

The response of the electrodes designated “Adsorption I” and “Adsorption II” dropped by 50% of their initial value on Day 10 and Day 7 respectively. On Day 14, their response was less than 35% of the original value. Weak binding and desorption might occur over a period of storage and analysis [24]. The loss in activity was greater than that of the biosensors obtained via crosslinking with glutaraldehyde. The enzyme immobilized onto the MMCHI membrane was found to be more stable compared to that immobilized on the LMCHI membrane. The response of the free GOD electrode dropped drastically by 95% on the second day and was less than 1% of its initial value after 10 days. The free enzyme-electrode was not suitable for reuse after one cycle.

The retention activity was influenced by the MW of chitosan. The gradual decrease of in the current might have been due to the temperature change experienced by the enzyme electrodes between the storage and experimental temperatures. Partial enzyme denaturation might have occurred over a period of time. The possibility of electrode fouling could also affect the sensitivity of the biosensors. The results of the GOD electrode suggest that the immobilization process permits the enzyme to be reused, resulting in operational stability over a period of time.

### Effect of Electroactive Compounds on Biosensor Response

Electroactive compounds present in the matrix can pose a problem in biosensors employing amperometric assays. The interference may be due to direct electrode oxidation with an increase in the anodic current or enzyme inhibition resulting in a reduced response current [25]. The selectivity of the biosensors therefore has to be evaluated in their presence.

The fourteen substances used to evaluate the selectivity of the two biosensors (GOD-MMCHI/PT and GOD-LMCHI/PT) included DL-glutamic, D(+) galactose, lactose, sucrose, oxalate, urea, citric acid, L-leucine, L-histidine, lactic acid, L-cysteine, ascorbic acid, acetaminophen and uric acid. The current obtained for each interfering substance at 0.1 mM was compared in the presence and absence of 5.0 mM glucose for the biosensors. No observable current shift in the presence of DL-glutamic, D(+) galactose, lactose, sucrose, oxalate, urea, citric acid, L-leucine, L-histidine and lactic acid. Hence, the concentration of these substances was increased to 0.5 mM in the presence of 5.0 mM glucose. Despite the increase in concentration, these substances did not affect the response of both biosensors in analyzing glucose. On the other hand, there were interfering signals from 0.1 mM acetaminophen (PCM), uric acid (UA), ascorbic acid (AA) and L-cysteine (Cys) when analyzing glucose at an applied potential of +0.6 V. This created a response deviation for the fabricated biosensors with easy co-oxidation of these substances at similar potentials [26]. The extent of interference encountered by both biosensors was not significantly different.

**Figure 9:**
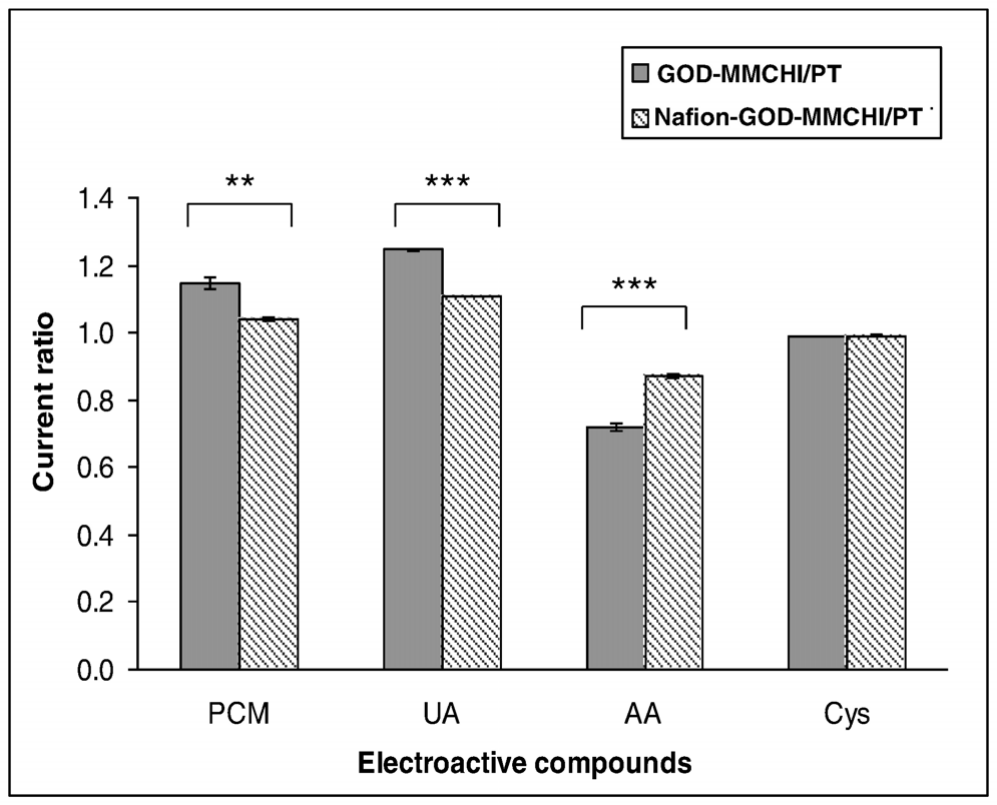
Ratio of currents for mixtures containing 0.1 mMelectroactive compound and 5.0 mM glucose to 5.0 mM glucose alone (mean±SEM, n = 3). ** and *** indicate significance level among the comparison groups at *P*<0.01 and *P*<0.001, respectively.

**Figure 10:**
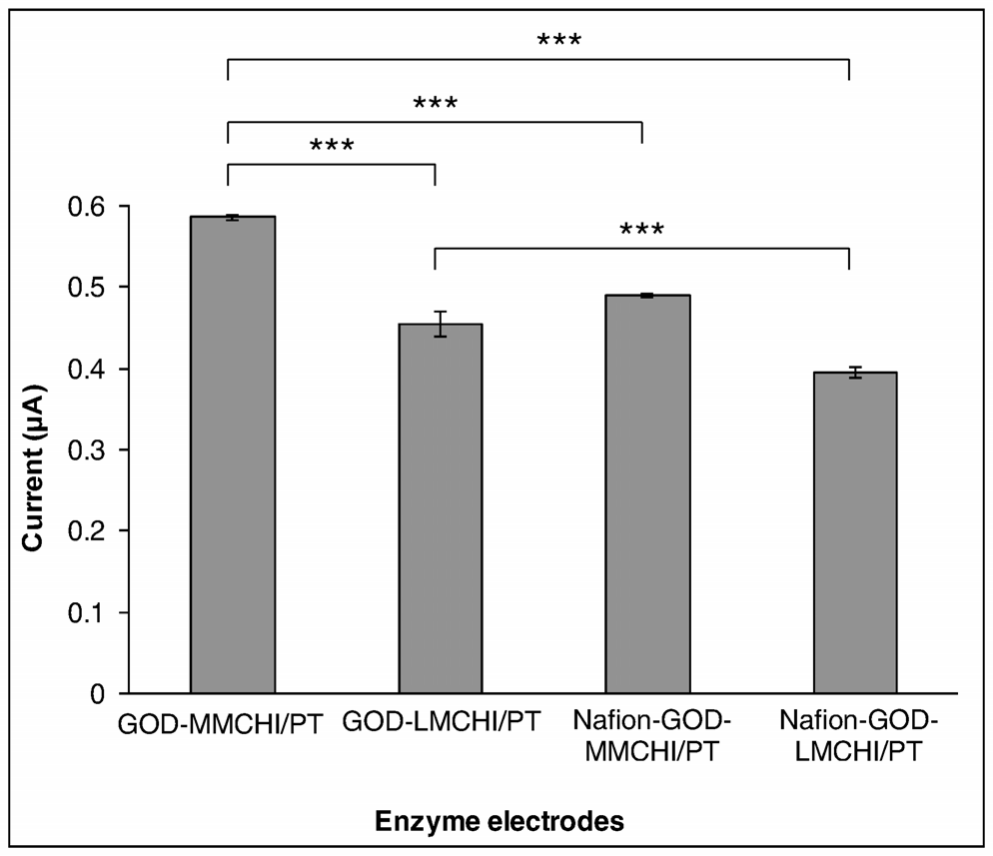
Ratio of currents for mixtures containing 0.1 mMelectroactive compound and 5.0 mM glucose to 5.0 mM glucose alone (mean±SEM, n = 3). ** and *** indicate significance level among the comparison groups at *P*<0.01 and *P*<0.001, respectively.

To overcome the effect of interferents, the biosensors were coated with a layer of Nafion to yield Nafion-GOD-MMCHI/PT and Nafion-GOD-LMCHI/PT. The effectiveness of this polyelectrolyte matrix to reduce the permeability of negatively charged substrates had been previously reported [27]. In addition, Nafion can prevent electrode fouling by proteins in the blood serum [28]. The presence of Nafion in Nafion-GOD-MMCHI/PT and Nafion-GOD-LMCHI/PT was found to reduce the effect of interference to different extents when compared to GOD-MMCHI/PT and GOD-LMCHI/PT (Table 3), because the negatively charged sulfonate groups in Nafion can prevent anions from partitioning onto the electrode surface [28], [29]. The deviation of current in the presence of PCM and UA sensed by Nafion-GOD-MMCHI/PT was significantly lower when compared with that of GOD-MMCHI/PT (Figure 9). There was no significant difference between the results obtained from GOD-LMCHI/PT and Nafion-GOD-LMCHI/PT in sensing these electroactive compounds. On the contrary, the response of Nafion-GOD-MMCHI/PT and Nafion-GOD-LMCHI/PT to glucose was significantly reduced (*P*<0.001) by about 17% and 13% respectively when compared to GOD-MMCHI/PT and GOD-LMCHI/PT (Figure 10). The addition of Nafion to the membrane acted as a diffusion barrier on top of the enzyme-chitosan membrane.

**Table 3:** Influence of some electroactive compounds on glucose biosensor response. Mean±S.E.M, n = 3.

Enzyme electrodesCompounds	Current ratios^r^			
	GOD-MMCHI/PT	GOD-LMCHI/PT	Nafion-GOD-MMCHI/PT	Nafion- GOD-LMCHI/PT
Acetaminophen	1.15±0.017	1.18±0.006	1.04±0.005	1.12±0.035
Uric acid	1.25±0.003	1.20±0.109	1.11±0.001	1.11±0.004
Ascorbic acid	0.72±0.013	0.73±0.004	0.87±0.005	0.81±0.006
L-cysteine	0.99±0.001	0.99±0.004	0.99±0.003	0.99±0.001

r Ratio of currents for mixtures containing 0.1 mMelectroactive compound and 5 mM glucose to 5 mM glucose alone.

In short, there was little interference encountered by the biosensors despite the absence of Nafion presumably due to the anti-interferents property of chitosan [29], [30]. In addition, GOD (pI  = 4.2) is negatively charged at pH 6.0 [31], [32] which can prevent anionic interferents from interaction with the surface of the platinum (Pt) electrode.

### Accuracy and Recovery

The glucose levels in rat serum samples were assayed using both glucose biosensors (GOD-MMCHI/PT and GOD-LMCHI/PT) and compared with those determined by an ABTS-based spectrophotometric method (Table 4).

**Table 4:** Comparison of glucose level in rat serum determined using glucose biosensors and ABTS-spectrophotometric method.

Sample no.	mConcentration of glucose (mM)			Concentration of glucose added (mM)	mConcentration of glucose found (mM)		mConcentration of glucose recovered (mM)		Recovery (%)	
	Spectrophotometric method	GOD-MMCHI/PT	GOD-LMCHI/PT		GOD-MMCHI/PT	GOD-LMCHI/PT	GOD-MMCHI/PT	GOD- LMCHI/PT	GOD- MMCHI/PT	GOD- LMCHI/PT
**1**	5.9472	5.9740	6.3997	4.9751	10.2994	11.3736	4.3254	4.9739	86.94	99.98
**2**	5.6908	5.1729	5.7066	4.9751	9.7295	10.6571	4.5566	4.9505	91.59	99.51
**3**	6.5721	6.0281	5.3659	4.9751	10.9061	10.1133	4.8780	4.7474	98.05	95.42
**4**	6.4751	6.9658	6.5908	7.4442	13.9230	13.9101	6.9572	7.3193	93.46	98.32
**5**	8.1342	8.5940	8.2489	7.4442	15.7004	15.4889	7.1064	7.2400	95.46	97.26

Recovery test using glucose biosensors is also shown.

m The average value of the three different measurements.

The accuracy of these biosensors was also evaluated by a standard addition method in determining the recoveries of glucose in rat serum samples. A comparison of the glucose levels in rat serum is given in Table 4. The results indicated that GOD-MMCHI/PT and GOD-LMCHI/PT exhibited reasonably satisfactory results with an average recovery of 93.10% and 98.10% respectively. The results therefore suggest that chitosan membranes could be used as a matrix in the development of glucose biosensors.

### Conclusion

There was anintermolecular interaction between chitosan and GOD. GOD-MMCHI possessed a higher catalytic activity than GOD-LMCHI. Immobilization via crosslinking exhibited a higher enzymatic activity than through adsorption. The immobilized enzyme-chitosan membranes were studied by incorporation into glucose biosensors. MMCHI possessed higher enzymatic activity and improved kinetic characteristics compared to that of LMCHI. GOD-MMCHI/PT exhibited higher sensitivity, repeatability, reproducibility, retention activity and stability. The response of the two biosensors to interferences, was almost similar. GOD-MMCHI/PT demonstrated better performance than GOD-LMCHI/PT for the determination of glucose in rat serum. In conclusion, chitosan membranes could be a suitable matrix in the development of glucose biosensors.

## Materials and Methods

### Preparation of Enzyme-chitosan Membranes

One gram of chitosan (Low molecular weight (LMCHI) and medium molecular weight (MMCHI)) was dispersed in 100 ml of acetic acid at concentration of 0.8% w/v (The physical properties of chitosan solution and membrane were studied by dissolving the chitosan in different organic acids (acetic acid, lactic acid and maleic acid). Both the chitosan samples were most soluble in aqueous acetic acid, followed by lactic acid and maleic acid). Chitosan membranes prepared from acetic acid were flexible, transparent, smooth and quick-drying. They exhibited good mechanical strength and elongation at break and the values were significantly higher than those prepared in lactic acid and maleic acid).The chitosan membranes were prepared into Petri dish with casting measurement 0.21 mL/cm^2^ membrane thickness for GOD-MMCHI/PT and 0.35 mL/cm^2^ membrane thickness for GOD-LMCHI/PT. After the membrane was formed, it was neutralized with 1% w/v sodium hydroxide (NaOH) for 30 minutes followed by rinsing with distilled water to remove excess NaOH. The neutralized membrane was cut into small squares (1.5×1.5 cm^2^) for immobilization. The extra membranes were kept in distilled water at 4°C until further use.

The glutaraldehyde activation method reported byMagalhães and Machado (1998) was adopted with a slight modification [33]. A smaller dimension of the chitosan squares (1.5×1.5 cm^2^) was used instead of 2.0×2.0 cm^2^. This was because the diameter of platinum electrode used was less than 1 cm. One side of the membrane square was coated with 20 µl of 1% v/v glutaraldehyde and allowed to dry at room temperature (25°C). Subsequently, 20 µl of glucose oxidase (GOD) (concentration ranges of 2.5 mg/ml to 40.0 mg/ml in 0.1 M of phosphate buffer, pH 7.0) containing 5% v/v glycerol was spread evenly onto the same surface of the membrane with the aid of a L-shaped glass rod. The immobilized membrane was left to dry at room temperature. The small amount of glycerol in the enzyme solution acts as an emollient to facilitate even spreading of GOD on the membrane surface.

For the adsorption method, (1.5×1.5 cm^2^) squares of chitosan membranes (MMCHI and LMCHI) were separately incubated in 1 ml of GOD solution (2.5 mg/ml) at 4°C for 24 hours. The membrane was then washed with distilled water and kept in phosphate buffer (0.1 M, pH 7.0) at 4°C until further use.

### Investigation of Intermolecular Interactions of Immobilized GOD-chitosan Membranes

Intermolecular interactions of chitosan membranes (MMCHI and LMCHI) with GOD were investigated by FTIR analysis. The FTIR spectra of GOD as potassium bromide (KBr) disks were obtained. Approximately 10 mg of GOD crystals and 100 mg of KBr was ground with an agate mortar and pestle for 5 min. About 40 mg of the mixture was then loaded onto an evacuable potassium bromide die and compressed for 1 min by applying an IR hydraulic press (Hydraulic Unit Model #3192, Carver Laboratory Equipment, Wabash, Indiana, USA) of 10 tons to obtain a yellowish, transparent and thin disk. It was placed in an oven at 40°C for 2 hours before analysis. GOD-MMCHI and GOD-LMCHI membranes (20 µl of 30 mg/ml GOD was used to crosslink onto chitosan membrane) were washed with distilled water to remove the unbound enzyme on the surface followed by air-drying at room temperature. They were kept in the oven at 40°C for 2 hours before scanning.

### Colorimetric Determination of Glucose

The activities of soluble and immobilized GOD were assayed using ABTS [2,2′-azino-bis(3-ethylbenzthiazoline)-6-sulfonic acid] method adapted from Bergmeyer and Bernt (1974) [34]. Reagent A was consisted of 0.1 M phosphate buffer (pH 7.0), 0.92 mM ABTS, 1.5 U/mL POD and 9 U/mL GOD, whereas reagent B was similar to reagent A but without GOD. For glucose calibration, the glucose concentration used was from 0.1221 mM to 1.0 M. For each run, 0.1 mL of mutarotated glucose solution was added to 5.0 mL of reagent A (oxygenated) and mixed using a vortex mixer. Initial reaction rate was determined by monitoring the rate of change of absorbance at 450 nm recorded by UV/VIS spectrometer (Lambda 45, Perkin Elmer Instruments, USA). The Michaelis-Menten constant for the soluble enzyme was determined following this procedure from Eadie-Hofstee plot (initial velocity of enzymatic activity, *v* versus 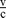, where *c* is concentration of glucose). For blood samples, 0.1 ml of serum was used instead of glucose standard. Thereafter, 0.1 mL of standard glucose solution (1.0 M) was added to 5.0 ml reagent B (oxygenated) and mixed well. For the determination of GOD activity, 20 µLof enzyme solution was added to reagent B. When GOD-immobilized membranes were used as samples, they were placed into reagent B for activity determination. In this case, the GOD membranes were sonicated for 1 hour before adding glucose solution. The glucose consumption was determined by the increase in absorbance at 450 nm.

The molar extinction coefficient (*E*) of FAD at 450 nm is 11.3±10^−3^ M^−1^cm^−1^ [35]. The absorbance (*A*) was converted to concentration, according to the equation *A* = *ELc*, where *L* is the cell thickness or path length of light through the sample (usually 1-cm) and *c* the concentration of absorbing material in the sample.

### Construction of the GOD-chitosan Electrode

**Figure 11:**
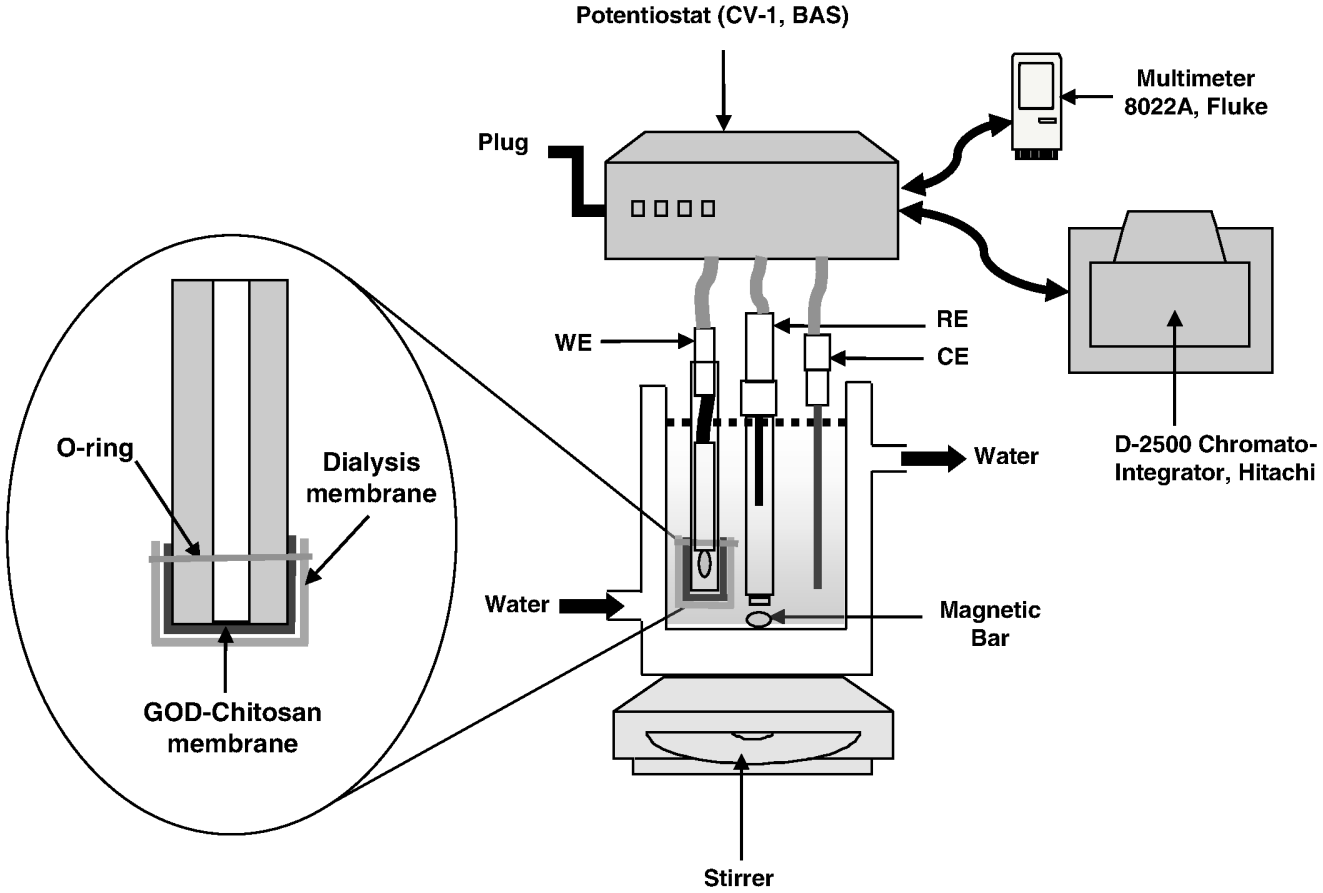
Schematic representation of the experimental set-up (WE: working electrode; RE: reference electrode; CE: counter electrode).

The platinum electrode was first polished with 0.05 µm alumina on a polishing pad, washed with distilled water and finally sonicated for few minutes to remove the alumina particles. The GOD-chitosan membrane placed over a moist dialysis membrane as a protective layer was fastened onto surface of the platinum electrode with an O-ring (Figure 11). Amperometric detection of glucose was performed using a potentiostat (cyclic voltammograph CV-1B, Bioanalytical Systems Inc. (BAS), West Lafayette, Indiana, USA) poised at +0.6 V connected to an integrator-plotter (Chromato-Integrator D-2500, Hitachi, Tokyo, Japan) and a digital multimeter (8022A, Fluke, USA). The experimental set-up for electrochemical measurements is illustrated in Figure 11. The conventional three electrodes consisted of a silver/silver chloride (Ag/AgCl) reference electrode (MF-2079, BAS, West Lafayette, Indiana, USA), a platinum wire (0.25 mm diameter, 99.99%, Aldrich, Milwaukee, Wisconsin, USA) as counter electrode and a platinum working electrode (MF-2013, BAS, West Lafayette, Indiana, USA) with the enzyme-chitosan layer. Unless stated otherwise, all experiments were carried out in 10 ml of phosphate buffer (0.1 M, pH 7.0) maintained at 25.0±0.1°C using a digital temperature controller (Model 9001, Poly Science, USA).

### Characteristics of the Glucose Biosensor

#### Response time

The response time of the glucose biosensor was determined from the time of the addition of the analyte to the time the anodic current produced achieved steady-state.

#### Calibration of glucose biosensor

Calibration of the glucose biosensors was performed under optimized experimental conditions. Aliquots of 10 µl of β-D-glucose stock solution were successively added into 10 ml of stirred phosphate buffer in an electrochemical cell. Three different glucose stock solutions at concentrations of 0.01 M, 0.1 M and 1.0 M were prepared to obtain the hydrodynamic response for glucose concentrations from 9.99×10^−6^ to 1.29×10^−1^ M. Calibration curves from the anodic current-glucose concentration plot for each biosensor were used to obtain the linear detection range.

#### Determination of apparent Michaelis-Menten constant

The apparent Michaelis-Menten constant (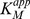) for the immobilized enzyme was determined from Eadie-Hofstee plot with the equation shown below:
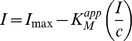
where *I* is the steady state current after the addition of substrate, *c* is the bulk concentration of the substrate (glucose) and *I*_max_ is the maximum current measured under saturated substrate condition. 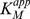
and the upper detection limit, *I*_max_ of the biosensor were then determined from the slope and intercept on the y-axis of the plot.

#### Repeatability and reproducibility

The repeatability generated by the glucose biosensors (GOD-MMCHI/PT and GOD-LMCHI/PT) was studied by measuring the anodic current generated by 3.98 mM glucose in 10 ml phosphate buffer (pH 6.0) for a total of 20 times in a single day. On the other hand, the reproducibility of the biosensors was studied by measuring the current generated by 3.98 mM glucose in 10 ml phosphate buffer (pH 6.0) by using six different glucose biosensors. The signals obtained were calculated and summarized as relative standard deviation (RSD). The RSD was calculated using the following equation:
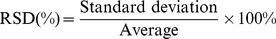



The stability of three types of biosensors was explored under optimal experimental conditions. The three types of biosensors were prepared via i) glutaraldehyde crosslinking (GOD-MMCHI/PT and GOD-LMCHI/PT), ii) physical adsorption method [Adsorption I (GOD-MMCHI) and Adsorption II (GOD-LMCHI)] and iii) coating the surface of the platinum electrode with 20 µL of GOD solution (30 mg/ml) at room temperature (25°C). The membranes were then placed onto surface of the platinum electrode covered with a layer of dialysis membrane and fastened with an O-ring.

The responses of GOD-MMCHI/PT and GOD-LMCHI/PT to 3.98 mM glucose were measured daily in triplicate for the first two weeks. After 2 weeks, the biosensors were tested every 3–5 days over a period of 60-days. The mean of the relative current to initial current sensed by these biosensors was plotted as function of time. The responses of the other two types of biosensors were measured under similar experimental conditions to compare the stability of these biosensors. All enzyme electrodes were kept in phosphate buffer and stored at 4°C when not in use.

#### Effect of electroactive compounds on biosensor response

The effect of possible interferences from electroactive substances were investigated by adding the interfering compounds of 0.1 mM to 0.5 mM into a standard solution of 5.0 mM glucose. The current obtained was then compared to that generated by glucose alone. A layer of Nafion (0.2% v/v diluted with alcohol) coated over the GOD-chitosan layer was prepared to study if the effect of interferences can be minimized or overcome.

#### Accuracy and recovery

Blood samples were collected intracardially according to the method reported by Orphan *et al*.(2003) from six non-fasting Sprague-Dawley malerats (∼250 g) aged between 3–5 months (Approval by Animal Ethic Committee, Universiti Sains Malaysia) [36]. The blood samples were allowed to coagulate for 1 hour after withdrawal and were then centrifuged at 3500 rpm for 10 min at 4°C. The supernatant (serum) was collected and glucose content was determined. In the electrochemical measurement, 200 µL of serum samples were added to 10 ml of 0.1 M phosphate buffer (pH 6.0) and the response was obtained at an applied potential of +0.6 V and temperature of 35°C. A separate analysis of glucose concentrations was carried out using the ABTS method based on spectrophotometric detection described under section “Colorimetric determination of glucose”. The recovery of glucose in serum was performed by adding known amount of glucose to the serum samples. The amount of added glucose was then determined using the two glucose biosensors, GOD-MMCHI/PT and GOD-LMCHI/PT. The concentration of glucose recovered was calculated from the difference in glucose concentration between the spiked and unspiked serum samples.

### Statistical Analysis

All the results were expressed as mean±standard error mean (SEM). The results were analyzed using one-way analysis of variance (ANOVA). When a statistically significant difference was obtained (*P*<0.05), LSD test was then performed. The relationship between parameters was analyzed using the Pearson correlation test.
